# Evaluation of Effective Chloramine-T Concentration to Be Incorporated in Dental Stone for Antimicrobial Activity

**DOI:** 10.7759/cureus.33200

**Published:** 2022-12-31

**Authors:** Kashish Mangal, Mithilesh M Dhamande, Seema Sathe, Rupali Patel, Chinmayee Dahihandekar

**Affiliations:** 1 Prosthodontics, Dr. DY Patil Dental College and Hospital, Pune, IND; 2 Prosthodontics and Crown & Bridge, Sharad Pawar Dental College and Hospital, Wardha, IND; 3 Prosthodontics, Mehta Dental Clinic, Mumbai, IND; 4 Prosthodontics and Crown & Bridge, Sharad Pawar Dental College and Hospital, Datta Meghe Institute of Medical Sciences, Wardha, IND

**Keywords:** antimicrobial therapy, antimicrobial, dental material, chloramine t, disinfection, type iv dental stone

## Abstract

Objective

The objective of this study was to determine the antimicrobial activity of type III gypsum at three different chloramine-T concentrations and to ascertain the most effective concentration to be added for optimum inhibitory activity against *Candida albicans*.

Method

Ten discs of type III gypsum were fabricated for each group. Standard type III gypsum without any disinfectant was used for the control group. For the experimental group, an admixture of chloramine-T and standard dental stone was employed in varying w/w concentrations (0.1%, 0.25%, and 0.5%). Discs were placed in a petri dish containing Sabouraud dextrose agar lawned with *Candida albicans* culture and incubated for 24 hours. The zone of inhibition created around the discs was measured and evaluated.

Result

The mean zone of inhibition (mean ± standard deviation) in the control group was 0 mm; 0.70±1.05 mm in group 1 (0.1% w/w concentration), 2.70 ± 2.35 mm in group 2 (0.25% w/w concentration), and 20.80 ± 1.68 mm in group 3 (0.5% w/w concentration). A one-way ANOVA test showed that there was a significant difference in the inhibition zone created around all groups (p < 0.05), with the discs of group 3 yielding the most positive results.

Conclusion

The addition of 0.5% chloramine-T to type III gypsum showed the most promising result, out of the concentrations tested, as a self-disinfecting dental stone and could be used for further investigations.

## Introduction

The realization of the need for disinfection to prevent cross-contamination is better understood and accepted by the population at large considering the current dire scenarios. Maintenance of the highest level of hygiene has become the new normal. This statement holds more relevance when dealing with dental procedures. It has been professed, proven, and reported that cross-contamination exists at various levels in any given dental procedure [1-6]. This can lead to occupational hazards for the clinician as well as the laboratory technician or an acquired infection for the patient [7]. Therefore, a multi-layered disinfection protocol is required to ensure the utmost prevention of dental cross-contamination.

Guidelines have suggested that both the dental impression of the patient and the subsequent cast retrieved from it should be disinfected by employing standard infection control protocols. But their factual application is often incorrect and inadequate. Many surveys and studies conducted by numerous researchers with geologically different sample populations evaluated the actual application of disinfection protocols in dental services and yielded a significant amount of data depicting the lack of optimum infection control practices [8,9]. This led to the development of multifarious dental materials with inherent disinfectant properties.

Type III gypsum, otherwise known as dental stone, is one of the most commonly used dental materials in the field of dentistry. It has a huge range of applications, from pouring impressions to being used in flasking procedures. In addition to being readily available, type III gypsum is also cost-effective. The most prominent usage of dental stone is in forming the positive replica of oral structures from dental impressions. As stated earlier, disinfection of such casts is eminent in combating cross-infection, so that no stone is left unturned to maintain the highest level of disinfection [1-3].

To disinfect the casts, numerous methods and materials have been suggested and reported. The disinfection can be done either at the time of model fabrication by incorporating disinfectant during the manipulation of gypsum [10-13] or by using spray [14,15] or immersion [16,17] techniques after the cast has been retrieved from the impression. All the methods have shown varying degrees of success as well as shortcomings of their own. The most significant flaws are the alteration of dental stone properties and the addition of an extra step in an already elaborated prosthetic workflow. Because of the laborious protocol along with a decreased awareness of the standards of infection control, a huge disparity is noticed in the prevention of cross-contamination [18,19]. Therefore, this study was undertaken to evaluate the effective concentration of the disinfectant, i.e. chloramine-T (CHT), which can be pre-mixed in powder form with the dental stone, enabling its usage as a self-disinfecting dental stone.

CHT (metabolite p-toluenesulfonamide) is a well-known disinfectant with usage in various fields like dental, medicine, agriculture, poultry, aquaculture, etc. Specific to dentistry, it has been reported to be experimentally used along with temporary restorative agents [20], as a storage medium for extracted teeth [21,22], as a disinfectant for elastomeric impression material [23], and as a dentifrice [24]. Even though CHT has been widely used for various purposes, its efficacy as a disinfectant product in addition to dental gypsum has not been explored. The literature on the usage of CHT, especially in its powdered form, in amalgamation with type III gypsum is very scarce; hence, this study was planned to explore new avenues in cast disinfection techniques.

## Materials and methods

This in vitro experimental study was performed with three experimental groups and a negative control group. A sample size of 30 (10 per group) was derived. The experimental groups had varied concentrations of chloramine-T trihydrate (Sigma Aldrich, St. Louis, Missouri, United States) in powder form mixed with type III gypsum (Kalstone, Kalabhai). Group 1 consisted of 0.10% (w/w) of disinfectant mixed with the dental stone. Group 2 had 0.25% (w/w) and group 3 had 0.5% (w/w) of CHT mixed with type III gypsum.

**Figure 1:**
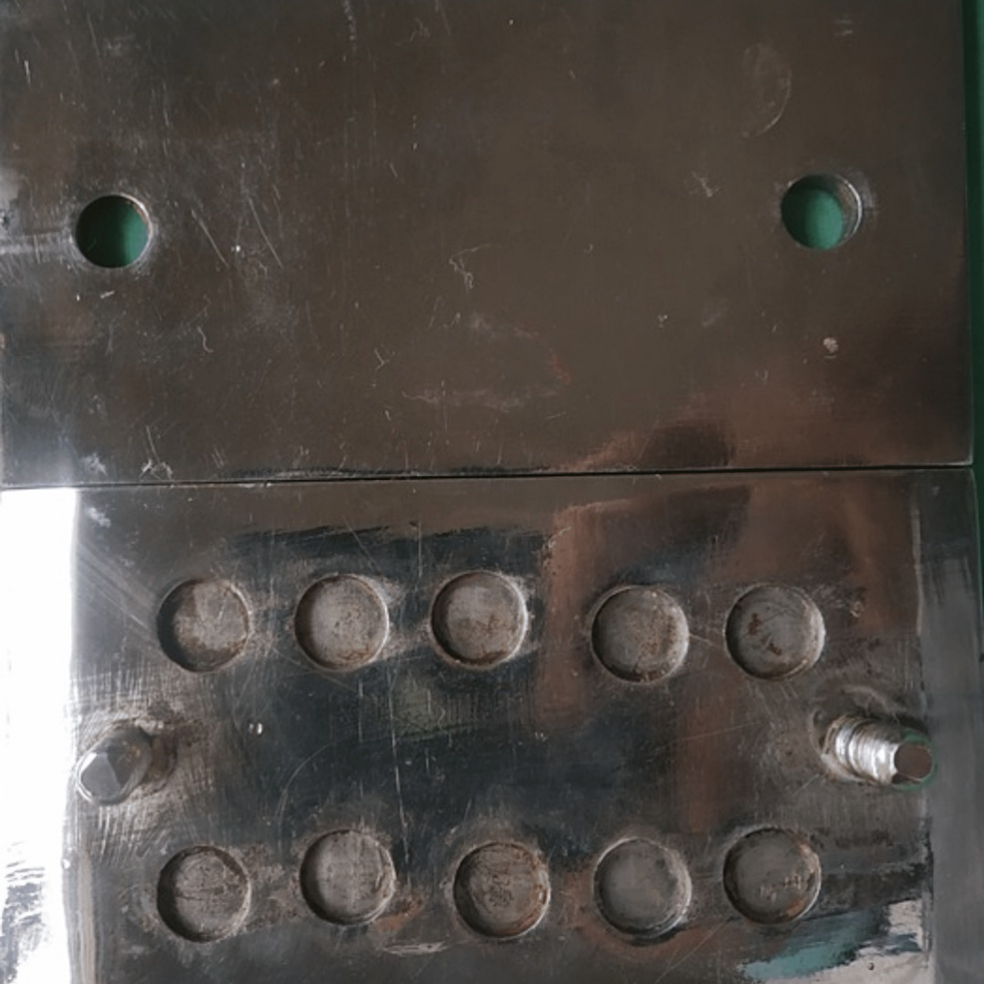
A custom split mold used for sample fabrication.

Samples were fabricated using a custom-made mold with 9 mm diameter and 3 mm thickness (Figure 1). To make the samples, both the disinfectant and gypsum product were weighed on a digital weighing scale (Sartorius, GmbH, Göttingen, Germany, model no. GC1603S-0CE). For each group, the dental stone was weighed up to 10 g and CHT was weighed according to the concentration selected (0.010 g for Group 1, 0.025 g for Group 2, and 0.050 g for Group 3). Each of the components was measured separately and then manually mixed according to its designated group.

For sample fabrication, the dental stone of each group was mixed according to the manufacturer's specified water-powder ratio, poured into the mold, and clamped. After 45 minutes, the mold was opened and samples were retrieved. Using a similar method, samples for all groups were fabricated. The sequentially numbered, opaque, sealed envelope (SNOSE) technique was used to ensure blinding in this in vitro study.

To test the antimicrobial efficacy, Kirby and Bauer method was used [25]. The zone of inhibition for each sample was tested against *Candida albicans* (*C. albicans*). A zone of inhibition is a halo that forms around the sample due to the inhibitory action of the disinfectant on the microbial culture. For the same, *C. albicans* was initially cultured in Sabouraud Dextrose Broth (SDA; HiMedia, Thane (West), Maharashtra, India). After turbidity was observed, it was compared against McFarland Standard (HiMedia) 0.5 for *C. albicans*. The suspension was then lawned evenly in three planes with a sterile cotton swab on a marked (for different groups) petri dish of SDA (HiMedia). Following this, samples of each group were placed using a pair of sterile forceps in their respective designated sections and then covered. A disc of unaltered dental stone was also kept with the test samples as a negative control. The entire assembly was then incubated for 24 hours.

**Figure 2:**
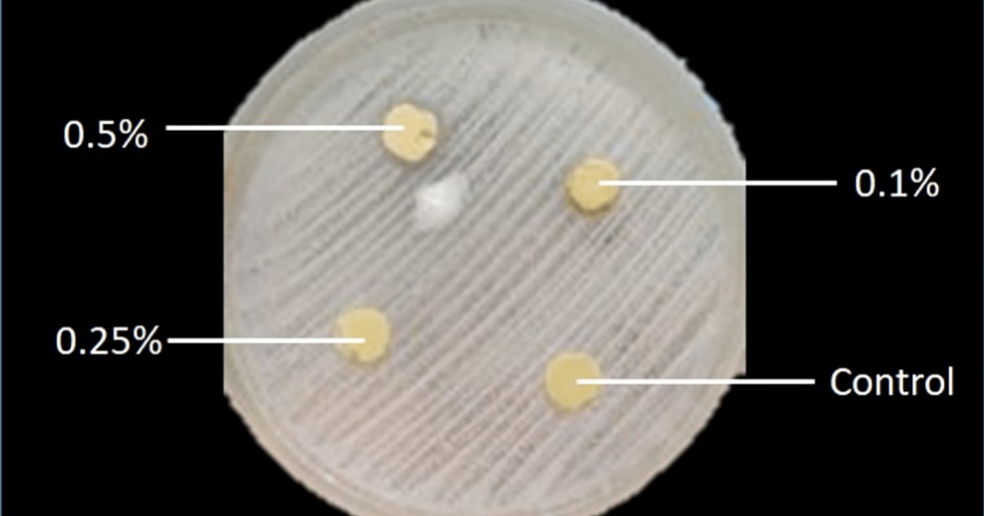
Petri dish after 24 hours of incubation. Notice the ring of the inhibition zone created around the sample of disc with 0.5% CHT in dental stone. Note: The white-streaked lines are of cultured *C. albicans.* CHT: chloramine-T.

After incubation, the cultured petri dishes were observed for the zone of inhibition (Figure 2). The halo zones were measured by a single researcher using digital calipers (accuracy up to 0.05 mm) along two perpendicular diameters. The average of the two measurements was taken as the statistical value and tabulated.

Normality of data was tested by Kolmogorov-Smirnov test, and therefore, statistical analysis was done by descriptive and inferential statistics using one-way ANOVA and multiple comparison Tukey test. IBM SPSS Statistics for Windows, Version 24.0 (Released 2016; IBM Corp; Armonk, New York, United States) was used in the analysis, and p<0.05 is considered a level of significance.

## Results

The mean zone of inhibition (mean ± standard deviation) in group 1 was 0.70 ± 1.05 mm, 2.70 ± 2.35 mm in group 2, and 20.80 ± 1.68 mm in group 3 (Table 1). By using a one-way ANOVA (F = 385.81, p = 0.0001), statistically significant variation was found in the mean zone of inhibition in samples of three groups. Table 2 shows the p-value of intergroup comparisons, group 1 and group 2 (p=0.047), group 1 and group 3 (p = 0.0001), and group 2 and group 3 (p = 0.0001).

**Table 1:** Mean zone of inhibition (in mm) of the tested groups. CHT: chloramine-T.

Group (concentration of CHT)	Mean ± standard deviation (in mm)
Group 1 (0.1%)	0.70 ± 1.05
Group 2 (0.25%)	2.70 ± 2.35
Group 3 (0.5%)	20.80 ± 1.68

**Table 2:** p-value derived on intergroup mean comparison showing significant difference using one-way ANOVA and multiple comparison Tukey test. *Significant result.

	Group 2 (0.25%)	Group 3 (0.5%)
Group 1 (0.1%)	0.047*	0.0001*
Group 2 (0.25%)	-	0.0001*

## Discussion

The need for an infection control program had been felt because a large number of bacteria, fungi, and viruses present in the dental environment were linked to debilitating and life-threatening diseases [5-7,26,27]. Every effort, therefore, must be made to avoid the cross-contamination of these microorganisms and to prevent the potential transfer of diseases in the dental setting. Direct physical interaction between the dental clinic and the dental laboratory is intrinsic to the practice of general dentistry. It is also one of those areas where maintenance of disinfection is the most difficult. Transmission of infected materials from various clinics to the laboratory not only places unwary staff at risk but also results in a high level of avoidable cross-contamination [1,3].

The prevention of contaminated dental impressions leaving the immediate chairside area or zone of contamination is an ideal situation. Several studies have shown that microorganisms can be recovered readily from stone casts separated from contaminated impressions [26]. Thus, numerous systems and protocols have been proposed to disinfect dental impressions or casts. Since it is said that prevention is always better than cure, an appropriate alternative is to decontaminate the cast produced from the impression by incorporating a disinfectant into the gypsum.

Type III gypsum was specifically used in this study due to its perennial use for making dental casts and their frequently overlooked disinfection. Thus, creating a self-disinfecting dental stone holds more importance. In everyday dental practice, type III gypsum is more frequently employed in comparison with other gypsum products. Even in specialized scenarios like preparing master casts of prepared teeth, the second pour-over die stone, or the replication of any maxillofacial moulage into a patient-like cast, the dental stone is preferred. This can be attributed to it being relatively cost-effective. Hence, type III gypsum was the material of choice for this study.

Table 1 helps in deriving that the mean zone of inhibition gave positive results in comparison with the negative control, thus confirming the potency of CHT as a disinfectant when incorporated in type III gypsum. From Table 2, it was found that 0.5% of CHT with type III gypsum was the highest among all tested concentrations. It can be said that group 3, i.e. 0.5% w/w concentration (Copyright registered, L-93269/2020, in the Copyright Office, Government of India) of CHT, when added to type III gypsum produced an efficient inhibition zone against *C. albicans*. This inference indicates that, with the addition of 0.5% w/w CHT, an optimally self-disinfecting dental stone could be produced. Because the samples in this study were fabricated with a pre-mix of the disinfectant and dental stone, no additional step of disinfection was required. The results obtained are in accordance with a study conducted by Schutt [10] in 1989 with a similar disinfectant. But unlike this study, the testing by Schutt was carried out to evaluate the turbidity produced instead of testing any specific microbe.

*Candida albicans* is one of the commensals in the oral cavity, with a varying presence ranging from 20% to 50% in healthy mouths [28,29] but exceeding a staggering figure of more than 70% in denture wearers [29,30]. However, in immunocompromised individuals, it is said that *C. albicans* has the potency to turn into a parasitic pathogen. Therefore, it was chosen for this study. Since no standards are currently existing regarding the dimensions of the observed inhibition zone with CHT against *C. albicans*, the presence or absence of the zone was considered as the baseline for the evaluation of the results. But to keep the testing as close as possible to that of any antifungal or antimicrobial drug testing, the discs were fabricated with 9 mm diameter and 3 mm width.

The results obtained only depict the existence of antimicrobial properties in the experimental admix of type III gypsum and CHT. A limitation of this research is that the testing was done only against *C. albicans;* thus, to prove the efficacy of the experimented compound, it should be studied against other microbes as well. An evaluation of the influence that this addition might have on the physical and mechanical properties of the dental stone is imperative. 

## Conclusions

This original research was done to evaluate if CHT could produce an antimicrobial effect, in conjunction with type III gypsum, at any of the tested concentrations. It was concluded from this study that 0.5% w/w concentration (copyrighted L-93269/2020) of CHT has an effective impact against *C. albicans*. Considering the results obtained, further research are warranted to test if this amalgamation could yield a probable self-disinfecting dental stone.
